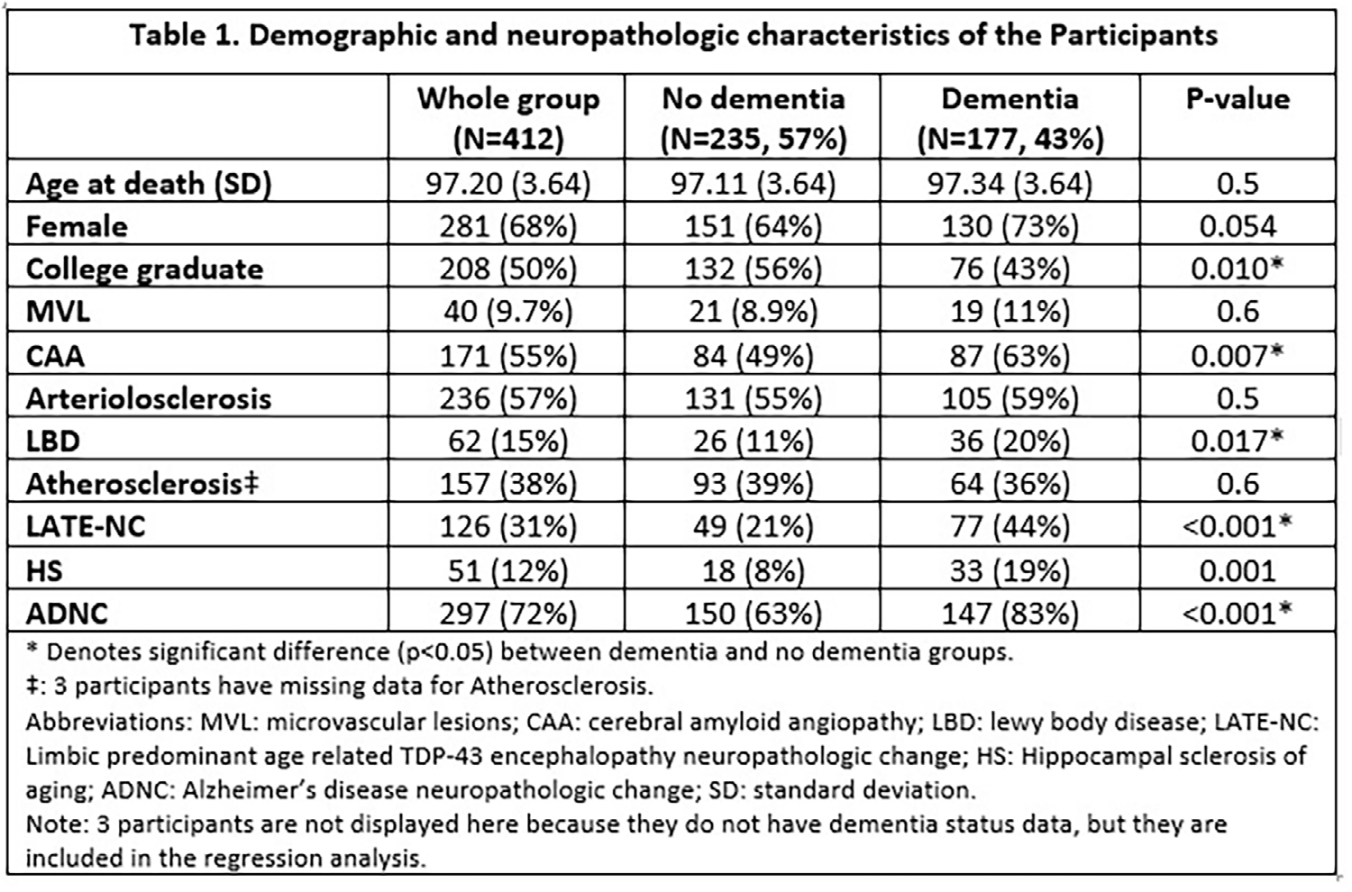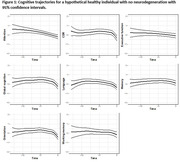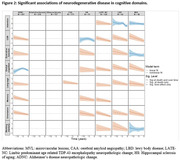# Contribution of neuropathological changes to cognitive decline in late life; The 90+ Study

**DOI:** 10.1002/alz.091777

**Published:** 2025-01-03

**Authors:** Jiaxin Yu, Davis C. Woodworth, Syed A. Bukhari, Ali Ezzati, Thomas J. Montine, Claudia H. Kawas, María M. M. Corrada, Tianchen Qian, S. Ahmad Sajjadi

**Affiliations:** ^1^ University of California, Irvine, Irvine, CA USA; ^2^ Stanford University, Stanford, CA USA

## Abstract

**Background:**

Cognitive impairment and dementia in late life are often due to co‐occurrence of multiple neuropathologic changes (NC) leading to multiple etiology dementia (MED). Our aim was to assess the contribution of these NCs by first studying the shape of cognitive trajectories in the absence of pathology and then to capture the association between longitudinal change in cognition and multiple common age‐related NCs in an oldest‐old cohort.

**Method:**

415 participants from The 90+ Study with longitudinal evaluations and autopsy data were included. The following criteria were used for NC presence: moderate or severe likelihood of Alzheimer’s disease NC (ADNC) according to NIA‐AA criteria; hippocampal or cortical limbic‐predominant age‐related TDP‐43 encephalopathy (LATE‐NC); presence of hippocampal sclerosis; limbic/neocortical Lewy bodies disease (LBD); moderate/severe scoring for cerebral amyloid angiopathy (CAA), atherosclerosis, and arteriolosclerosis, and ≥2 microvascular lesions (MVLs). We considered longitudinal scores of the clinical dementia rating sum of boxes (CDR‐SB), global cognition measured by mini mental state examination (MMSE), and composite scores for 6 cognitive domains (Figure 1). We quantified the time‐varying relationship between each of clinical and cognitive measures, as outcome, and the neuropathologic changes, as independent variables, using varying‐coefficient mixed effects models that can identify nonlinear relationships and account for within‐subject repeated measures. The models were adjusted for age at death, sex, and education.

**Result:**

Table 1 summarizes the demographic and neuropathologic characteristics of the participants. Mean age at death was 97.2, 68% were female, and 50% were college graduate. Most NCs were significantly more frequent in the dementia group. Figure 1 shows the trajectories of CDR‐SB and various cognitive domains in a hypothetical individual with no NCs. Attention and executive function showed early decline while CDR and cognitive domains showed an NC independent decline around five years before death. Figure 2 demonstrates the significant associations of NCs with decline in CDR‐SB and cognitive domains. LATE‐NC was the strongest predictor of CDR and cognitive outcomes closely followed by ADNC, HS, LBD, and MVL.

**Conclusion:**

Our results demonstrate the contribution of various NCs to trajectories of cognitive outcomes in the oldest old and highlight the importance of non‐Alzheimer’s NCs in this age group.